# IS-98-ST1 West Nile Virus Derived from an Infectious cDNA Clone Retains Neuroinvasiveness and Neurovirulence Properties of the Original Virus

**DOI:** 10.1371/journal.pone.0047666

**Published:** 2012-10-23

**Authors:** Céline Bahuon, Philippe Desprès, Nathalie Pardigon, Jean-Jacques Panthier, Nathalie Cordonnier, Steeve Lowenski, Jennifer Richardson, Stéphan Zientara, Sylvie Lecollinet

**Affiliations:** 1 UMR 1161 VIROLOGIE ANSES-INRA-ENVA, Agence nationale de sécurité sanitaire de l’alimentation, de l’environnement et du travail (ANSES), Maisons-Alfort, France; 2 Unité des Interactions Moléculaires Flavivirus-Hôtes, Department of Virology, Institut Pasteur, Paris, France; 3 Mouse functional Genetics, Institut Pasteur, Paris, France; 4 Unité d’Histologie et d’Anatomie Pathologique, Ecole Nationale Vétérinaire d’Alfort, Maisons-Alfort, France; Duke-NUS Graduate Medical School, Singapore

## Abstract

Infectious clones of West Nile virus (WNV) have previously been generated and used to decipher the role of viral proteins in WNV virulence. The majority of molecular clones obtained to date have been derived from North American, Australian, or African isolates. Here, we describe the construction of an infectious cDNA clone of a Mediterranean WNV strain, IS-98-ST1. We characterized the biological properties of the recovered recombinant virus in cell culture and in mice. The growth kinetics of recombinant and parental WNV were similar in Vero cells. Moreover, the phenotype of recombinant and parental WNV was indistinguishable as regards viremia, viral load in the brain, and mortality in susceptible and resistant mice. Finally, the pathobiology of the infectious clone was examined in embryonated chicken eggs. The capacity of different WNV strains to replicate in embryonated chicken eggs closely paralleled their ability to replicate in mice, suggesting that inoculation of embryonated chicken eggs could provide a practical *in vivo* model for the study of WNV pathogenesis. In conclusion, the IS-98-ST1 infectious clone will allow assessment of the impact of selected mutations and novel genomic changes appearing in emerging European strains pathogenicity and endemic or epidemic potential. This will be invaluable in the context of an increasing number of outbreaks and enhanced severity of infections in the Mediterranean basin and Eastern Europe.

## Introduction

The zoonotic West Nile virus (WNV), which belongs to the *Flavivirus* genus (family *Flaviviridae*), circulates in natural transmission cycles involving avian hosts and ornithophilic mosquito *Culex* ssp, whereas horses and humans are regarded as dead-end hosts [1], [2], [3]. Arthropod-borne WNV can infect the central nervous system (CNS) of various host species and neurological disease is a grave, albeit infrequent, complication of WNV infection [4]. Once inside the CNS, WNV infects neurons and gives rise to severe immunopathology. Although asymptomatic in the majority of cases, WNV infection has been associated with severe meningo-encephalitis and acute flaccid paralysis in humans.

WNV contains a positive single-stranded RNA genome of about 11,000 nucleotides comprising a single open reading frame flanked by two untranslated regions (UTRs) at the 5′ and 3′ ends. Genomic RNA codes for a single and long polyprotein which is co- and post-translationally cleaved by cellular and viral proteases. There are three structural proteins (C, prM/M and E) followed by seven nonstructural (NS) proteins (NS1, NS2A, NS2B, NS3, NS4A, NS4B, NS5) [5], along with NS1’, which results from a -1 ribosomal frameshift event [6]. Structural proteins provide the structural elements of viral particles, while nonstructural proteins play a role in viral replication, virion assembly, and evasion of host antiviral immune responses [7], [8], [9], [10], [11].

WNV is maintained in endemic cyles in Africa, West Asia, Russia, India and Europe, and more recently in North America [3]. Phylogenetic analysis has permitted identification of lineages 1 (divided into clades 1a to 1b) to 5 [12], [13]. Lineage 1 WNV strains have been reported in many regions, including Africa, Europe, the Middle East, Russia, India, and North America since 1999 [3]. WNV subsequently spread to South America [14]. Since the mid-1990’s, epidemics with a high incidence of neurological disease and death have occurred in Eastern Europe and the Mediterranean area, *e.g*. in Israel in 1998, and more regularly in North America since 1999 [1], [3]. WNV emergence in North America has also been associated with significant mortality in birds, a phenomenon that had not been previously reported [1].

WNV transmission in Europe involves strains that belong to the South Europe/Kenyan and the Israeli/American clusters of lineage 1a, or to lineage 2 [15], [16]. Lineage 1 has been involved in most of recent WNV epidemics in the world. However, analysis of molecular determinants of virus virulence has mainly concerned North American isolates with a particular focus on NY99 strains [17], [18]. In contrast, few studies have been conducted to elucidate the virulence of WNV isolates from Europe/Middle East in common hosts such as horses and avian species [19].

During the 1998 epidemic in Israel, WNV strain IS-98-ST1 was isolated from a stork with severe neurological symptoms [20]. IS-98-ST1 is suitable for the study of viral determinants of WNV virulence, as well as host factors involved in viral pathogenicity [21], [22], [23], [24], [25]. To date, a few molecular clones derived from North American, African and Australian strains of WNV are available for the study of viral neuropathogenicity [1], [3], [9], [26], [27], [28]. We report here that WNV strain IS-98-ST1 recovered from an infectious cDNA clone reproduces the pathobiological properties of the parental strain, and is thus well-suited for studying the virulence of WNV isolates from Europe/West Asia. Also, the pathobiology of the novel WNV infectious clone and other WNV strains was examined in embryonated chicken eggs. The capacity of different WNV strains to replicate in eggs closely paralleled their growth properties in mice, suggesting that inoculation of embryonated chicken eggs could provide a practical *in vivo* system for the study of WNV pathogenesis.

## Results

### Construction of a European/Mediterranean Infectious Clone

**Figure 1:**
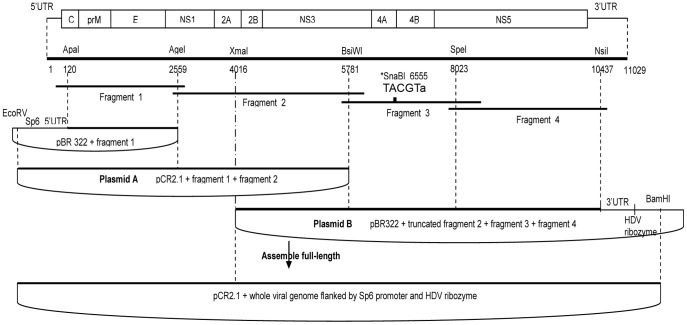
Schematic representation of the cloning strategy. The flavivirus genome is represented approximately to scale. Unique or rare restriction sites used for cloning and their location (numbering based on the sequence from Genbank no. AF481864) are shown at the top. Four cDNA fragments represented by thick lines were synthesized from IS-98-ST1 viral genomic RNA by RT-PCR to cover the complete coding WNV genome. The cDNA fragments were first cloned into the pCR2.1 plasmid, and then digested using unique restrictions sites and subcloned into the destination plasmid. The full length infectious clone was obtained by the extraction of fragment 2+3+4 from plasmid B after an enzymatic digestion with *XmaI* and *BamHI* followed by its insertion into plasmid A downstream of fragment 1+2. One silent mutation (shown in lower case) was engineered in the pCR2.1+ fragment 3 plasmid to create a *SnaBI* site.

WNV strain IS-98-ST1 is a highly pathogenic isolate originating from a sick stork during the 1998 epidemic in Israel [20]. Its ssRNA+ genome was amplified into four pieces for generating the infectious clone construct. After RT-PCR amplification of viral RNA genome, each of the four amplicons was subcloned in pCR2.1, a high-copy vector used for the cloning of RT-PCR products. Propagation of bacteria at room temperature allowed maintenance of intact inserts in pCR2.1. Difficulties in the sequential insertion of each fragment in pBR322 led us to devise an alternative two plasmid strategy (Fig. 1). Fragment 1 (nt 120–2559) was inserted in pBR322 and transferred along with the SP6 promoter upstream of fragment 2 (nt 2560–5781) to pCR2.1 (Fig. 1). This construction will be referred to as plasmid A. Fragment 4 (nt 8023–10473) and then fragment 3 (nt 5782–8022) were inserted into pBR322. A 1740 bp sequence at the 3′ end of fragment 2 was inserted upstream of fragments 3 and 4 within pBR322. This construction will be referred to as plasmid B. The sequence encompassing fragment 2 to fragment 4 was excised from plasmid B, and inserted downstream of fragments 1 and 2 in plasmid A. A *SnaB*I restriction endonuclease site was introduced into the NS4A gene within fragment 3 and further used as a genetic marker. The resulting plasmid was introduced in *E.coli* DH5α strain. It carries the full-length cDNA from WNV IS-98-ST1 in a single plasmid and was designated WNV-infectious clone (IC) cDNA. The stability of the clone was evaluated by propagating the DH5α *E.coli* hosting the full-length IC cDNA plasmid for six continuous passages as described in the paper published by Shi *et al.* [3]. Resulting plasmids were digested with *AvaI*, and displayed the same digestion pattern (data not shown).

### The RNA Transcript of the WNV-IC cDNA Clone was Highly Infectious

**Figure 2:**
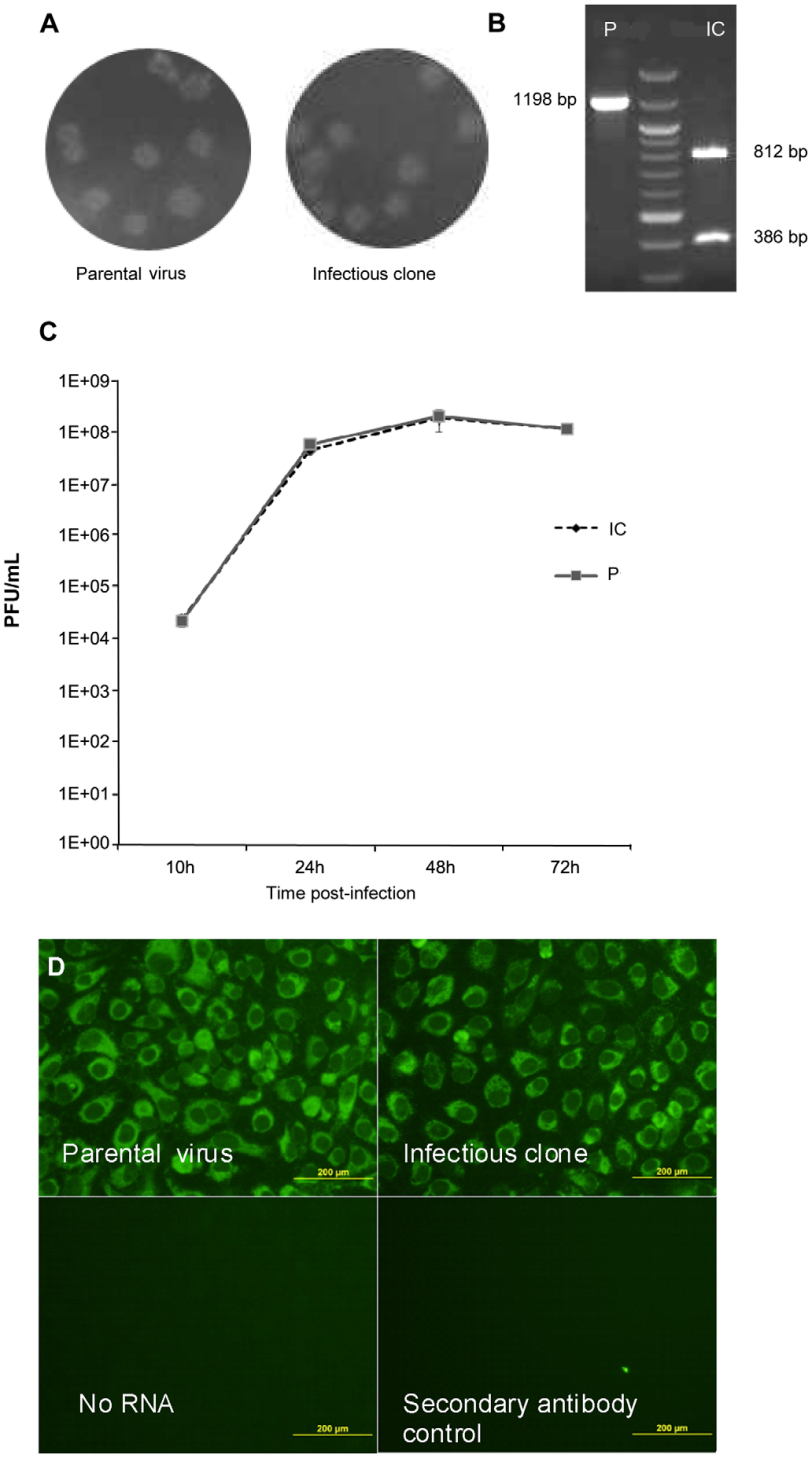
*in vitro* validation of the biological properties of the IC virus. (a) Plaque morphology of parental and IC-derived WNV on Vero cells. Vero cells in six-well plates were infected with 100 PFU of parental or recombinant virus. Plaques were visualized 3 days post-infection by staining with crystal violet. Data shown are representative of two independent experiments. (b) *SnaBI* digestion profile of the 1198 bp RT-PCR (5743F–6941R) fragment amplified from parental or IC-derived WNV stocks. (Quick Load 1 kb DNA Ladder, New England Biolabs). (c) Growth kinetics of parental (P) and infectious clone (IC) viruses in Vero cells at an MOI of 1. At the indicated time post-infection, culture supernatants were collected and viral titers were determined by plaque assay on Vero cells. Error bars represent the standard deviation of triplicates. (d) Immunofluorescence with anti-NS1 antibodies on Vero cells infected at an MOI of 1 with either the parental or IC-derived WNV 3 days post-infection. Data shown are representative of two independent experiments.

*In vitro* transcription of WNV-IC cDNA generated a transcript of ∼ 11,000 nt. Transcripts were used to electroporate Vero cells. Vero supernatants were harvested at day 2 post-infection (p.i) in order to minimize emergence of quasi-species and used to infect new Vero cells (P1). Viral titers in supernatant obtained for P1 reached 7×10^7^ Plaque Forming Unit (PFU)/ml. The parental virus was used as a positive control in the titration experiments. A similar plaque morphology was observed for parental and IC viruses (Fig. 2 (a)). IFA was performed to check for the antigenicity of viral particles, and intracellular NS1 was detected for both parental and recombinant viruses (Fig. 2 (d)).

### WNV Recovered from the cDNA Clone Retained its Genetic Marker

To exclude the possibility that recovery of virus from transfected cells resulted from contamination by the parental virus, we tested the presence of the *SnaB*I restriction site in RT-PCR products amplified from the virus produced in the transfected Vero cells (Fig. 2 (b)). The parental virus genomic cDNA was not digested by *SnaBI*. In contrast, the IC virus PCR product generated fragments of 812 bp and 386 bp following *SnaB*I digestion, indicating that the virus recovered from the transfected cells was derived from infectious full-length RNA from the WNV-IC plasmid.

### Identification of Reversion Mutation during Cloning

Every intermediary construct and the final IC plasmid were entirely sequenced. A G3401A mutation was identified in the WNV-IC plasmid that changed a UGG (Trp) codon into a UAG stop codon within the NS1 coding region. This mutation must have been positively selected during plasmid amplification in *E. coli*. Nevertheless, and surprisingly, the mutation was absent from the recovered WNV genome. That is, the sequence of packaged RNA genomes was identical to the expected IS-98-ST1 genomic sequence. To test whether the recovery of infectious RNAs was due to recombination with wild-type virus, a truncated transcript was designed that did not encompass the region bearing the restriction endonuclease site *SnaB*I in the corresponding cDNA. This truncated transcript was used to transfect Vero cells. A cytopathic effect (CPE) was seen 3 days after transfection in cells transfected with the full-length WNV-IC RNA transcript. In contrast, no CPE was observed up to 7 days post-transfection with the truncated transcript. In addition, quantitative RT-PCR on total RNA extracted 7 days post-transfection with primers specific for the 3′NC region detected no truncated viral RNA (data not shown). Therefore, infectious particles recovered from the transfection of Vero cells with WNV-IC transcripts were not the product of a recombination event.

### Parental and Recombinant WNV Displayed Indistinguishable Replicative Capacities

To test the viral replication rate, Vero cells were infected with WNV parental or IC virus (Fig. 2 (c)). At 48 h post-infection (p.i.), CPE was observed in Vero cells infected with either virus. Viral RNA amounts were higher during the first 24 hours. However, at later stages, 48 h and 72 h p.i., viral RNA amounts decreased due to increasing cell death (data not shown). Infectious virus recovered from cell supernatants noticeably increased in the first 24 h p.i. and peaked at 48 h p.i (Fig. 2(c)). Growth curves exhibited no significant difference between the WNV parental and IC viruses. Therefore, the two viruses were indistinguishable with regard to replication in mammalian cells.

### Parental and Recombinant WNV Displayed the Same Virulence, Pathogenicity and Capacity for Neuroinvasion in vivo

**Figure 3:**
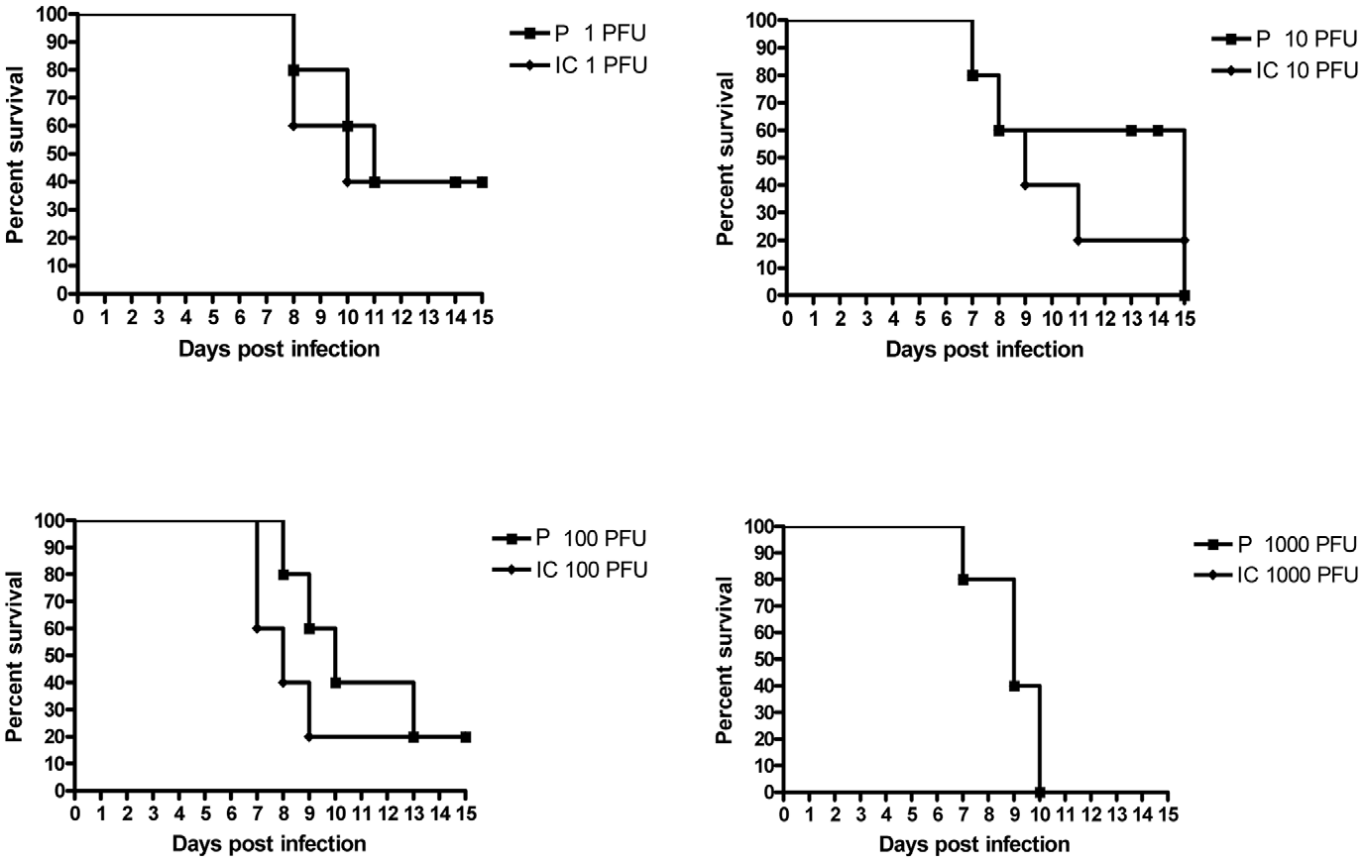
*in vivo* validation of the biological properties of the IC virus in susceptible mice, survival curves. Groups of 5 adult female BALB/c outbred mice were injected i.p with 1, 10, 100, 1000 PFU of parental (P) or recombinant (IC) IS-98-ST1 virus. Mice were monitored for 15 days post-infection. Data shown are representative of two independent experiments.

**Figure 4:**
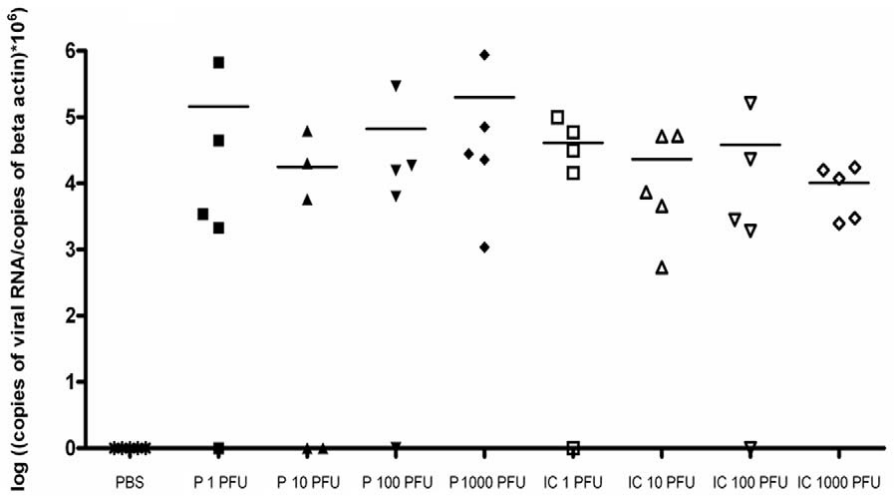
*in vivo* validation of the biological properties of the IC virus in susceptible mice, viremia. Viral RNA copy number in blood of BALB/c outbred female mice (n = 5) injected i.p with 1, 10, 100, 1000 PFU of parental (P) or recombinant (IC) IS-98-ST1 virus, 3 days post-infection. Quantification was performed in duplicate.

**Figure 5:**
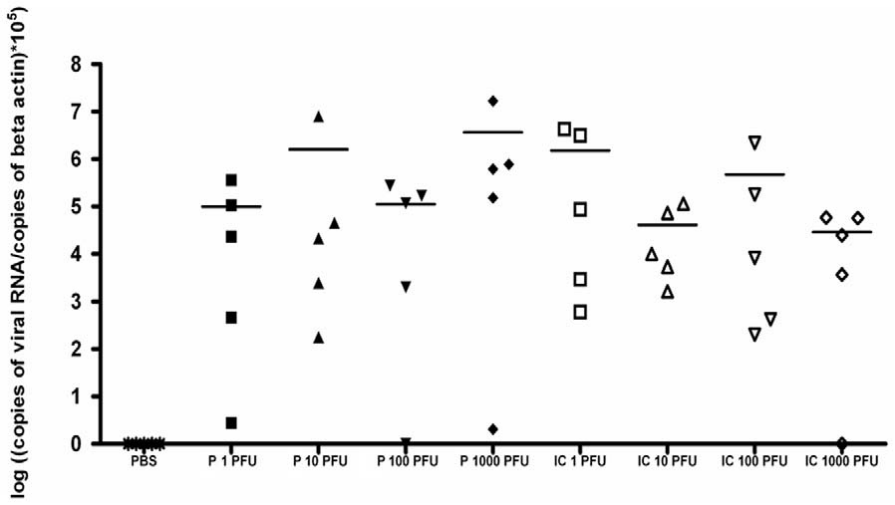
*in vivo* validation of the biological properties of the IC virus in susceptible mice, viral load. Viral RNA copy number in brains of BALB/c outbred female mice (n = 5) injected i.p with 1, 10, 100, 1000 PFU of parental (P) or recombinant (IC) IS-98-ST1 virus. Quantification was performed in duplicate.

**Figure 6:**
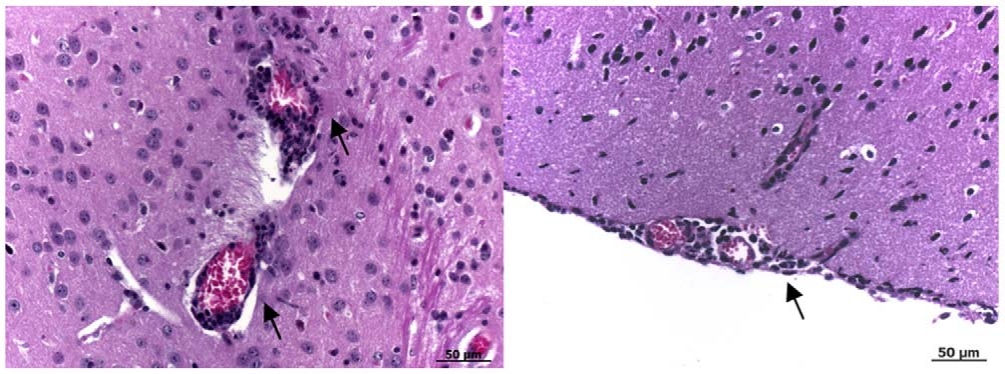
*in vivo* validation of the biological properties of the IC virus in susceptible mice, histology. Histology performed on mouse brain 7 days p.i revealed inflammatory lesions in the meninges characterized by diffuse lymphocytic or lymphoplasmocytic infiltrates, characteristic of lymphocytic meningitis (right panel, arrows). Lymphocytic perivascular cuffs were also visible in the brain parenchyma, indicative of encephalitis (left panel, arrows). These lesions were observed in two of four animals or in one mouse for the infectious clone and the Israeli strain, respectively. Tissue sections were stained with hematoxylin, eosin and saffron.

Conventional laboratory mice develop encephalitis and die after inoculation by the peripheral route [21]. To compare the virulence of the WNV parental and IC viruses, adult BALB/cJ mice were inoculated intraperitoneally (i.p.) with 1, 10, 100 or 1000 PFU. No control mice died consecutive to PBS injection. The survival curves did not differ significantly at each dose of infection for parental and IC viruses with death occurring between days 7 and 13–15 (Fig. 3). These data agree with previous reports [22], [29]. We measured the viremia at day 3 p.i. and the viral load in the brain at time of death. There was no significant difference in the viremia at day 3 p.i and in the viral load in the brain (Fig. 4 and 5). The *SnaB*I endonuclease restriction site was present in the cDNA of the WNV-IC virus recovered from infected mice, thus confirming that the mice were indeed infected with the recombinant virus (data not shown). In addition, inflammatory lesions were observed in the brain, within the meninges. They were characterized by diffuse lymphocytic or lymphoplasmocytic infiltrates, typical of lymphocytic meningitis, while some lymphocytic perivascular cuffs were also visible in the brain parenchyma, revealing encephalitis (Fig. 6). No lesions were observed in PBS-treated mice. Thus, the virulence of WNV parental and IC viruses was indistinguishable in susceptible BALB/cJ mice.

**Figure 7:**
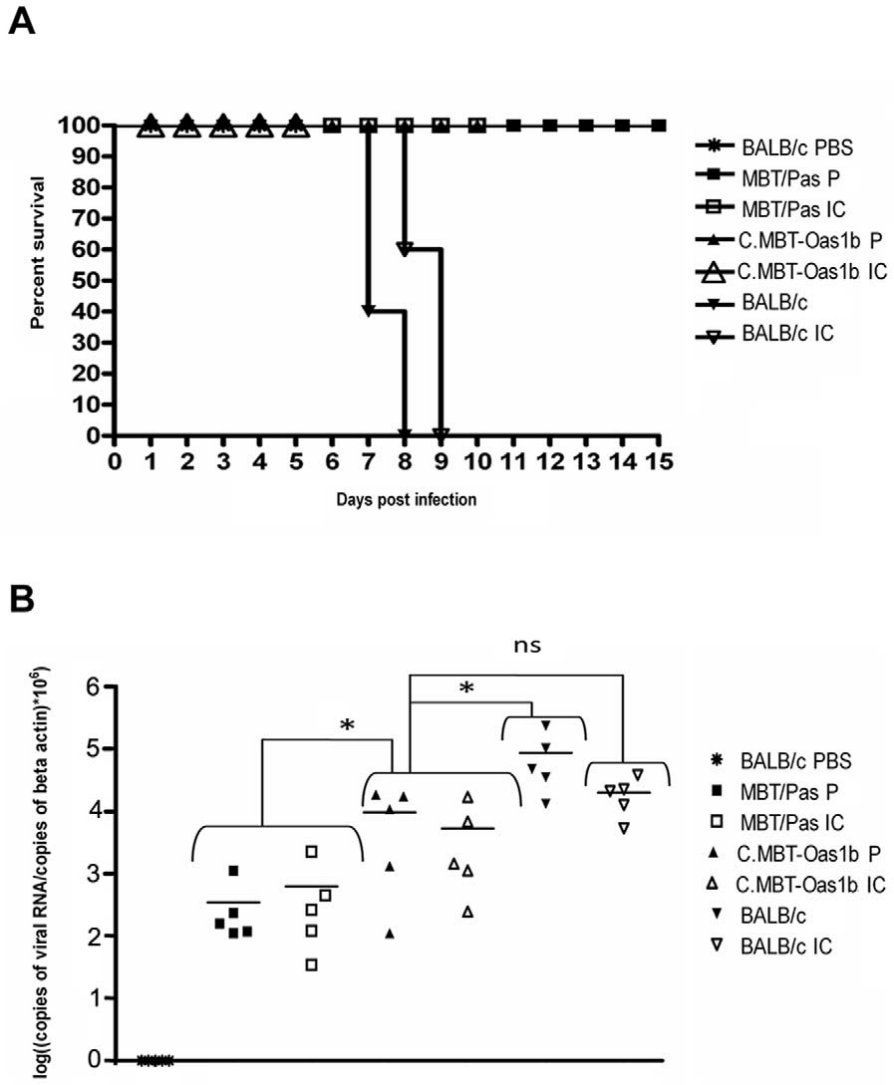
*in vivo* validation of the biological properties of the IC virus in resistant mice. Groups of 5 adult female or male outbred MBT/Pas, congenic C.MBT-Oas1b or BALB/c mice were injected i.p. with 1000 PFU of parental (P) or recombinant (IC) IS-98-ST1 virus. The mice were monitored for 15 days p.i. (a) Survival curves and (b) viral RNA copy number in blood of mice 3 days p.i. * shows significant differences between groups. ns: not significant.

To exclude the possibility that the susceptibility of BALB/c mice may have concealed differences in the virulence of the parental and cloned virus, we examined whether the WNV parental and IC viruses were equally avirulent in resistant inbred strains of mice. Indeed, Mashimo *et al.* [21] established that susceptibility of BALB/c mice to infection with WNV strain IS-98-ST1 correlates with a premature stop codon within exon 4 of the oligoadenylate synthetase 1b (*Oas1b*) gene. Mice expressing a full-length OAS1B protein are resistant to infection with WNV strain IS-98-ST1 [21], [25]. To test the virulence of WNV parental and IC viruses in resistant hosts, we took advantage of MBT/Pas and C.MBT-*Oas1b* mice. MBT/Pas mice derive from *Mus m. musculus* progenitors; they carry a wild-type allele at the *Oas1b* locus. C.MBT-*Oas1b* mice carry the *Oas* gene cluster of the MBT/Pas mice within a BALB/c genetic background. MBT/Pas and C.MBT-*Oas1b* express a full-length OAS1B protein and are resistant to infection with WNV [25]. MBT/Pas, C.MBT-*Oas1b* and BALB/c mice were inoculated with 1000 PFU of WNV parental or IC virus. All BALB/c mice died within 9 days p.i. In contrast, all MBT and C.MBT-*Oas1b* mice survived the infection (Fig. 7 (a)) independent of the WNV stock. Viremia at day 3 p.i was measured by qRT-PCR. Fig. 7 (b) shows that viral RNA was detected in the blood of all infected mice. Viral RNA levels were not significantly different in mice infected with either the WNV parental or IC virus. However, viral RNA levels were significantly higher in susceptible BALB/c mice than in resistant MBT/Pas and C.MBT-*Oas1b* mice. Interestingly, viral RNA levels were significantly lower in MBT/Pas mice than in C.MBT-*Oas1b* mice, indicating that viral replication is lower in MBT/Pas mice than in C.MBT-*Oas1b* mice during the first days p.i. This suggests that MBT/Pas mice might carry resistance alleles that are not present in the C.MBT-*Oas1b* congenic mice.

### Parental and Recombinant WNV Displayed the Same Virulence and Capacity for Neuroinvasion in an Embryonic Avian Model

**Figure 8:**
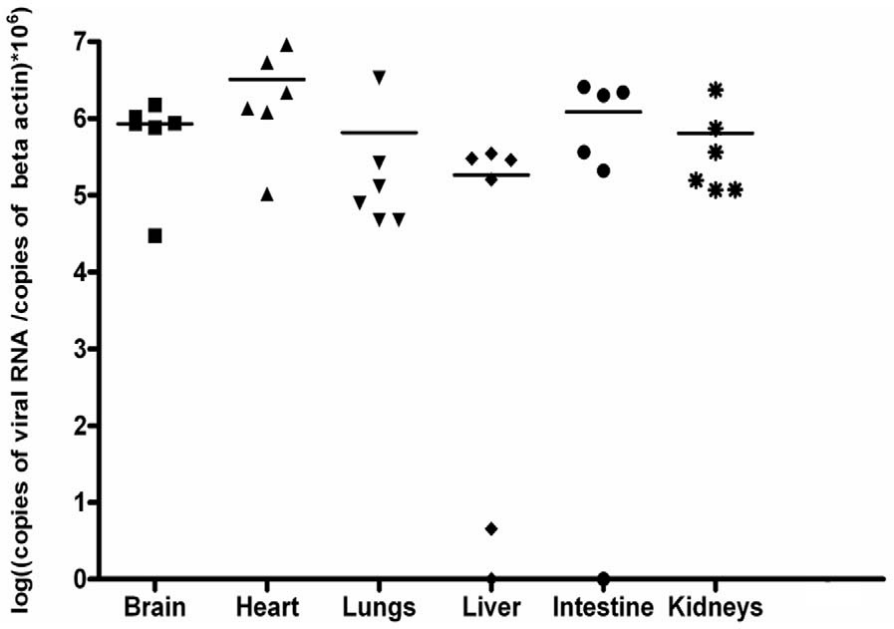
Viral load in different organs of chicken embryo infected with IS-98-ST1 WNV. Groups of 6 ten-day-old pathogen-free chicken eggs were infected with 1 PFU of IS-98-ST1 parental virus via the intra-vascular route. Viral load in different organs was quantified by quantitative RT-PCR. PBS controls were negative for all organs.

Birds are the principal amplifying hosts for WNV. Previous studies have shown that American crows and chicken embryos are susceptible to WNV infection [30], [31]. In experiments conducted by Crespo *et al*. [31], WNV was recovered from embryonated chicken eggs after inoculation with organ extracts from wild birds. Here, we evaluated the embryonated chicken model as a practical avian-based system to compare the virulence of WNV strains. To test the susceptibility of chicken embryos to infection, eggs (N = 6) were inoculated intravascularly with 0.1, 1, 10 or 100 PFU of the WNV IS-98-ST1 parental strain. The mortality was monitored for 7 days. Similar curves were obtained for the infectious doses of 1, 10 and 100 PFU, with 100% of embryos dying between day 4 and 5 p.i (Fig. S1). However, eggs infected with 0.1 PFU exhibited a 50% lethality rate. The 1 PFU dose (ten-fold the fifty percent lethal dose [LD50]) was chosen for the following experiments. The brain, heart, lung, liver, intestine and kidney are targets for WNV in birds [32], [33]. Therefore, the viral loads were measured in these organs. No significant differences were observed between the viral loads in these organs (Fig. 8).

**Figure 9:**
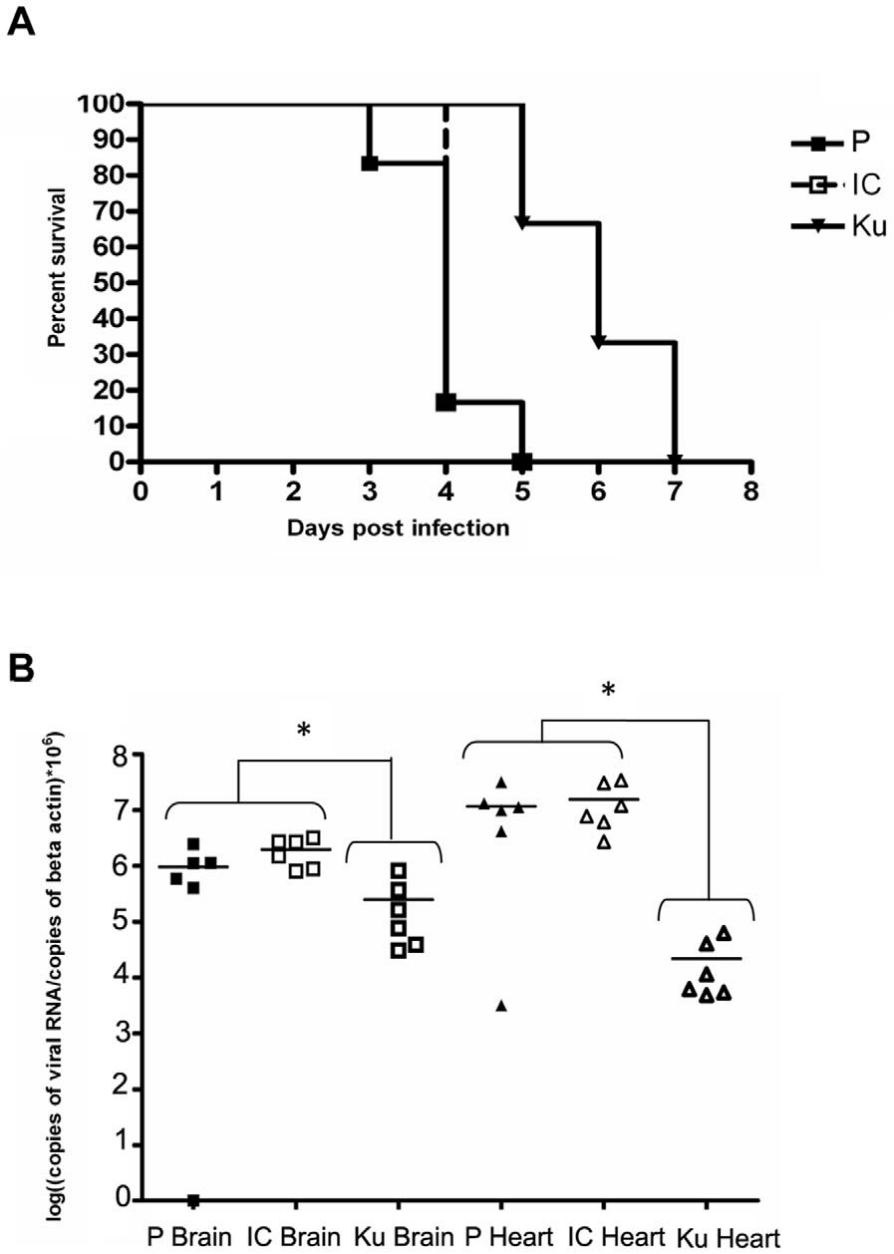
*in vivo* validation of the biological properties of the IC virus in chicken embryo. Groups of 6 ten-day-old pathogen-free chicken eggs were infected with 1 PFU of either parental (P) or recombinant (IC) IS-98-ST1 or Kunjin (Ku) virus by the intra-vascular route. (a) Survival curves and (b) viral RNA load in brains and hearts at day 4 p.i, quantified by quantitative RT-PCR, are shown. PBS controls were negative for all organs. Data are representative of two independent experiments, except RNA quantification at day 4 p.i for Kunjin, which was performed once. * shows significant differences between groups.

We chose to focus our analysis on the brain and the heart. Mortality and viral load in these tissues were monitored in groups of 6 eggs (Fig. 9). The WNV strain Kunjin served as a low pathogenic control [34]. A delay in mortality was observed in eggs infected with the Kunjin strain, with death occurring between days 4 and 7. When eggs were infected with either the parental strain or the IC virus, death was massive at day 4. The mortality rate was not significantly different in eggs inoculated with the WNV parental or the IC virus (Fig. 9 (a)). We measured the viral load in the brain and heart at day 4 p.i. The viral load in these tissues was not significantly different in the eggs infected with the parental strain or its cognate IC (Fig. 9 (b)). By contrast, the viral load was significantly lower in the eggs infected with Kunjin strain. Interestingly, similar macroscopic lesions appeared on the skin (petechia) and hemorrhages were observed in the brain, heart, kidneys and liver at the time of death for all viruses tested. Altogether these data show that the virulence of WNV parental and IC virus was indistinguishable in this avian model.

## Discussion

We report the generation of a full-length cDNA derived from the highly virulent Mediterranean WNV strain IS-98-ST1 that permitted recovery of a fully replicative virus. The establishment of a reverse genetic system for WNV is essential for the study of viral neurovirulence and neuroinvasiveness. Infectious clones allow introduction of genetic modifications and facilitate identification of the molecular determinants of viral virulence. Nevertheless, cDNA constructs comprising the full flavivirus genome have been widely reported to be unstable in prokaryotic expression systems [1], [3], [26], [35]. To avoid constructs all together, genome-length RT-PCRs have been employed, but pose the risk of generating a heterogeneous RNA population, owing to mutations occurring during RT-PCR or to viral diversity in the original virus stock. Different approaches have thus been used to improve the stability of full-length flavivirus cDNA clones [3]. Introns have been inserted to interrupt genes encoding products that may be toxic in bacteria [8], [36], and relatively permissive *E.coli* strains have been identified. Full-length flavivirus cDNA from NY99, B956 and Kunjin WNV strains have previously been cloned in plasmids amplified in the HB101 *E.coli* strain [3], [8], [26]. In the present study, we employed a plasmid cloning strategy to construct an infectious clone of the IS-98-ST1 WNV strain (WNV-IC). Selection of the appropriate plasmid is critical [3], [37]. The pBR322 plasmid was chosen because it had been used successfully to generate West Nile infectious clones in the past [3], [8], [26]. Plasmids encoding flavivirus sequences downstream of cytomegalovirus (CMV) or lac promoters displayed high instability due to expression of products that were toxic for *E.coli* [38]. To avoid such problems we selected the promoter for the bacteriophage SP6 RNA polymerase for *in vitro* transcription of viral RNA [8], [26], [39], [40]. An additional G nucleotide was inserted downstream of the promoter to ensure more efficient transcription initiation [26]. To this end, the pBR322 plasmid was modified (GeneCust, Dudelange, Luxemburg) to incorporate the SP6 promoter, the WNV 5′UTR (from position 1 to 97), part of the gene encoding the WNV capsid (from position 97 to 120) and the WNV 3′UTR (from position 10437 to 11029), along with the hepatitis δ virus ribozyme (HDVR). The HDVR mediated termination of WNV transcripts at their natural 3′ end, thereby ensuring removal of nonviral sequences downstream of the WNV 3′UTR that might have interfered with the initiation of viral RNA replication [26], [41]. Although infectious clones have been generated in the past, they were found to comprise mutations with respect to their parental virus or have been very difficult to handle [3], [8], [26], [42]. As a consequence, many of the infectious WNV clones currently in use have been obtained in two pieces [1], [30], [43]. Our infectious clone was constructed as a single piece and ensures propagation of recombinant virus. It also displayed remarkable stability, even after numerous passages in bacterial hosts (data not shown).

A G3401A mutation was identified in the NS1 coding region in the WNV-IC plasmid, as well as plasmid B, that changed a UGG (Trp) codon into a UAG stop codon. When it appeared during cloning, the fragment encoding the 3′ end of the NS1 open reading frame (ORF) could be stably inserted in frame of the fragment coding the 5′ end, thus reconstituting the complete NS1 ORF. Moreover, repeated efforts to correct the stop mutation by site-directed mutagenesis failed, even though parallel mutagenesis of other areas of the IC plasmid succeeded. This suggests that this change might stabilize the recombinant plasmid by neutralizing the leaky expression of NS1 in *E. coli*. The toxicity of the NS1 protein in *E. coli* is supported by previous observations made in other laboratories [1], [43]. Nevertheless, we observed that the stop codon is spontaneously corrected at an early time point after RNA transfection and that viral genomes recovered from newly formed viral particles carried the fully corrected gene. Thus, the molecular clone described in the present study supports efficient generation of viral particles bearing the fully intact genome of the parental virus. It is interesting to note that a nonsense mutation was observed in the NS1 ORF at a nearby position (C3342A) in a plasmid bearing an IC derived from the NY99 isolate [30]. This unnatural stop codon was not, however, detected in the genome of viral particles that were subsequently recovered, in which a A3342T plasmid-to-virus mutation had actually restored the original cysteine residue. Kinney *et al.* [30] hypothesized that a T7 polymerase transcription error had substituted the correct nucleotide. As such a reversion was observed twice in our hands and four times by others, we suggest that a more complex molecular mechanism may account for this event, such as translational stop codon readthroughs that have been described for viral genomes, for RNA viruses in particular [44], [45], [46], and that could explain the obtention of viral particles from an RNA transcript with a premature stop codon.

Crucially, the replicative and pathogenic properties of parental and recombinant viruses were indistinguishable in murine and avian *in vivo* models. Thus, the recombinant virus may be used to identify molecular determinants of virulence. Embryonated chicken eggs constitute a convenient *in vivo* system, and have previously proved to be a practical tool for the amplification of low doses of WNV [31]. In the present study, we show for the first time that chicken embryos can be used as an avian model to compare the virulence of WNV strains. The differences in the replication rate between Kunjin (attenuated strain) and IS-98-ST1 (highly virulent) parental or WNV-IC virus were observed here in the context of the embryonic avian immune system [47], [48]. The embryonated chicken egg model has been shown to be useful to study the virulence of various bacteria and fungal pathogens [49]. Our results strongly suggest that this model may also be exploited to investigate viral virulence.

Infectious clones of lineage 2, African strain B956 [26], lineage 1, American NY99 [1], [3], [30], [36], [43], [50], and Australian Kunjin strains [8], [40] have been generated and used to decipher the role of viral proteins in virulence [43], [51], neuroinvasiveness [1], [36] and escape from host defences [23], [52], as well as to evaluate the impact of point mutations [23], [53], [54]. These tools have substantially facilitated identification of molecular determinants of WNV virulence, albeit in a non-European context. WNV epidemiology differs between Europe and North America, in that outbreaks in Europe are more limited in space and duration. It is presumed that at least part of the difference stems from genetic divergence between North American and Mediterranean viral isolates. It is thus important to extend molecular studies of WNV to European strains, for which results have sometimes been at odds with those for the NY99 strain. Brault *et al.* [54] identified a point mutation, T249P, in NS3, as playing an important role in the virulence of NY99 in American crows, while Sotelo *et al.* [55] showed that a 2007 Spanish strain bore the same mutation but was much less virulent than the NY99 strain in mice. Clearly, comparison of molecular clones derived from different continents is indispensable for identifying the genetic differences underlying the different epidemiological patterns.

Finally, the IS-98-ST1 infectious clone will be useful to assess the impact of selected mutations or novel genomic changes appearing in emerging European strains, in the context of increasing numbers of outbreaks and severity of infection in the Mediterranean basin and Eastern Europe.

## Materials and Methods

### Cell Lines and Viruses

Vero cells, ATCC® CCL-81, were grown in Dulbecco’s Modified Eagle Medium (D-MEM, Gibco) supplemented with 5% fetal bovine serum (FBS) (Lonza), and 1 mM sodium pyruvate. 1 U/ml of penicillin and 1 µg/ml of streptomycin were added to all media (Invitrogen). Cells were maintained in CO_2_ incubators (5% CO_2_) at 37°C. The Israeli isolate IS-98-ST1 of WNV has been described elsewhere [20]. Briefly, IS-98-ST1 was isolated from the cerebellum of a stork in Israel in 1998 on Vero cells and the recovered virus was used to infect mosquito AP61 cells. A third-passage stock of IS-98-ST1 on AP61 cells was used in the following experiments. WNV Kunjin virus (KUN) was provided by the National Reference Center for Arboviruses at the Pasteur Institute, Paris.

### Plaque Assay

7×10^5^ Vero cells were seeded into each well of a 6-well plate and infected with parental virus or infectious clone for 1 h 30 min at 37°C. The cells were overlaid with 2% seaplaque agarose (Lonza) in MEM (Gibco) containing 5% FBS (Fetal Bovine Serum), 1% sodium pyruvate, 1 U/ml of penicillin and 1 µg/ml of streptomycin. 72 h post-infection, cells were fixed with 4% paraformaldehyde and stained with 0.4% crystal violet for 24 h in a humid chamber at 37°C.

### cDNA Synthesis and Cloning

Vero cells were infected with a multiplicity of infection (MOI) of 1 to 10 with parental IS-98-ST1 WNV, and virus was harvested from cell culture media at 72 h postinfection. Genomic RNA was extracted with the RNeasy kit (Qiagen). cDNA fragments covering the complete viral coding genome were reverse transcribed from genomic RNA by using Superscript III (Invitrogen) followed by PCR with Phusion Taq polymerase (New England Biolabs) according to the manufacturers’ instructions. In particular, four genomic fragments were amplified using primers 95F 5′-CGA TGT CTA AGA AAC CAG GAG- 3′, 2585R 5′-GTG GCG TTT CAG GGT AAT AC- 3′ for fragment 1, 2539F 5′-GAT GTG GAG GCT TGG ATG GAC- 3′, 5804R 5′-CAT TTT GGG TAC TCC GTC TCG-3′ for fragment 2, 5743F 5′-CGT GCT GGA AAG AAA GTA GTC-3′, 8082R 5′-GTA GAA CAC ATC CAC TCC ACTC-3′ for fragment 3, 7975F 5′-GAA GTC AGA GGG TAC ACA AAG G-3′, 10552R 5′-CAG CAC CGT CTA CTC AAC TTC-3′ for fragment 4. They were first subcloned into the pCR2.1 plasmid and then cloned into pBR322 following enzymatic digestions using standard procedures [56]. All restriction endonucleases were purchased from New England Biolabs. Sequences were verified after cloning.

The chemically competent *E.coli* bacterial strain DH5α (Invitrogen) was used as the host for construction and propagation of WNV-IC cDNA clone. Standard cloning procedures were followed [57], except that transformed bacteria were propagated at 25°C.

### Sequencing

Nucleotide sequencing was performed by Eurofins MWG Operon (France).

### RNA Transcription and Transfection

Plasmid WNV-IC, containing the full-length cDNA of WNV flanked by the Sp6 promoter and Hepatitis δ virus (HDV) ribozyme, was amplified in *E. coli* DH5α and purified with the Miniprep kit (Qiagen). For *in vitro* transcription, 1 µg of WNV-IC plasmid was linearized with *BamHI*. The digestion reaction mix was purified with the QIAQuick purification Kit and the linearized plasmid was quantified by spectrophotometry. The Sp6 MEGAscript High yield transcription kit (Ambion) was used to *in vitro* transcribe 1 µg of DNA in a 20 µl reaction mixture in the presence of 4 mM Cap Analog (New England Biolabs). The reaction mixture was incubated at 37°C for 4 h, followed by the addition of DNase I for 45 min at 37°C to remove the DNA template. RNA was stored at –80°C.

For transfection, the 20 µl (∼ 20 µg) RNA transcript mix was used to electroporate 6×10^6^ Vero cells resuspended in 0.4 ml cold phosphate-buffered saline (PBS), pH 7.4, in 0.4 cm cuvettes with the GenePulser apparatus (Bio-Rad); the following settings were applied: 4 pulses of 0.29 kV and 25 µF at 4s intervals. After a 10 min recovery, cells were mixed with D-MEM supplemented with 2% FBS and incubated in a T-25 flask (5% CO_2_ at 37°C) until a cytopathic effect (CPE) was observed (referred to as passage 0, P0). A 1 ml aliquot of P0 tissue culture supernatant at day 2 was used to infect a new flask of Vero cells (referred to as passage 1, P1) and incubated until a CPE was observed. Virus was harvested as tissue culture supernatant, clarified by centrifugation at 600 g for 5 min, stored in aliquots at −80°C, and designated as infectious clone derived virus (IC virus).

### Genetic Marker Analysis of the Recombinant and Parental Viruses

An *SnaBI* restriction site was inserted into the cDNA using the QuickChange Site-Directed Mutagenesis Kit (Stratagene) as a silent genetic marker in order to distinguish recombinant progeny virus from the corresponding parental virus, by substitution of an A for a T at position 6555 in the viral cDNA. RNA was extracted from recombinant virus harvested from supernatant on day 3 post-transfection and parental virus by using the RNeasy kit (Qiagen). A 1198 bp fragment including the genetic marker was amplified by RT-PCR from RNA extracted from either recombinant or parental virus with primers 5743F 5′- CGT GCT GGA AAG AAA GTA GTC-3′ and 6941R 5′- CTG CTT ATG TCA CTC TTG GTC TTA TC-3′. The RT-PCR products were purified using the QIAQuick PCR purification protocol (Qiagen), and then digested with *SnaBI* and analyzed by electrophoretic migration on a 1% agarose gel. When the *SnaBI* restriction site is present, digestion of the PCR product generates fragments of 812 and 386 bp.

### Replication Curves

Subconfluent Vero cells seeded in 12-well plates were inoculated with either parental or WNV-IC derived virus diluted in D-MEM at an MOI of 1 in quintuplet wells. Following incubation for 1 h 30 min in 5% CO_2_ at 37°C, viral inoculum was removed. Monolayers were washed twice with fresh D-MEM, and 1 ml of complete D-MEM was added to each well. The plates were incubated for up to 3 days in 5% CO_2_ at 37°C. At 10, 24, 48, and 72 h time points, medium was removed. At the indicated time post-infection, culture supernatants were collected and viral titers were determined by plaque assay on Vero cells.

### Immunofluorescence

Expression of viral NS1 protein in cells infected at an MOI of 1 was monitored by immunofluorescence (IFA) analysis with murine IgM antibodies directed against the WNV NS1 protein (Millipore) and with a fluorescein isothiocyanate-conjugate of goat anti-murine IgM antibody (Abcys), as described elsewhere [58]. Fluorescence was monitored under a fluorescence microscope equipped with a video documentation system (Zeiss).

### Quantitative RT-PCR

RNA was reverse-transcribed and amplified using conditions described by Linke *et al.* [59] using the AgPath-ID One-Step RT-PCR Kit (Applied Biosystems), with primers WNproC-10F 5′-CCTGTGTGAGCTGACAAACTTAGT-3′/WNproC-153R 5′-GCGTTTTAGCATATTGACAGCC-3′ in the 5′ untranslated region (UTR) or primers WN3′NC-F 10538 5′-GAGTAGACGGTGCTGCCTGC-3′/WN3′NC-R 10627 5′-CGAGACGGTTCTGAGGGCTTAC-3′ in the 3′UTR for amplification of viral RNA and primers ACTB-966F 5′-CAGCACAATGAAGATCAAGATCATC-3′/ACTB-1096R 5′-CGGACTCATCGTACTCCTGCTT-3′ for amplification of cellular RNA, and probes WNproC 5′-FAM-CCTGGTTTCTTAGACATCGAGATCT-TAMRA-3′ or WN3′NC 10564c 5′-FAM-ACCCAGTCCTCCTGGGGT-MGB-3′, or ACTB1042-67 5′-VIC-TCGCTGTCCACCTTCCAGCAGATGT-TAMRA-3′, respectively. Primers and probes were used at 0.4 and 0.2 µM concentrations, respectively. Reaction mixtures (25 µL) contained 5 µL RNA and all samples were analyzed in duplicate. Amplification was performed in an AB 7300 Real-Time PCR system (Applied Biosystems). The thermal profiles of the reaction were as follows: 45°C for 10 min (RT), 95°C for 10 min (Taq activation), and 40 cycles at 95°C for 15s and 60°C for 1min (amplification).

For quantification, quantified WNV RNA and β-actin RNA were used as a viral standard and to standardize RNA extractions, respectively. WNV RNA was obtained from viral cultures on Vero cells. β-actin RNA was kindly provided by the FMDV French Reference Laboratory (ANSES, Maisons-Alfort).

### Virulence in Mice

Mice were housed in an environmentally controlled room under biosafety level 3 conditions and were given food and water *ad libitum*. Female outbred BALB/c mice (Charles River Laboratories, L’Arbresle, France) and MBT/Pas and C.MBT-*Oas1b* mice provided by Jean-Jacques Panthier (Pasteur Institute, Paris) were obtained at 5 weeks of age and were acclimatized for 1 week. All mice were 6 weeks of age at the start of the experiment. 5 mice per group were inoculated intraperitoneally (i.p.) with diluent alone (PBS, endotoxin free, pH 7.4) or with 1, 10, 100 or 1000 PFU of parental virus or infectious clones thereof. Mice were evaluated clinically for 2 weeks. Observed clinical signs included ruffled fur, paresis, hind leg paralysis and tremors. Blood was collected in EDTA at day 3 post-infection (p.i), and the presence of viral RNA was confirmed by quantitative RT-PCR [53] after RNA extraction with the QIAamp viral kit (Qiagen). Brains were recovered from mice shortly after their death, and the presence of virus was confirmed by quantitative RT-PCR after RNA extraction with the RNeasy kit (Qiagen). Control mice were euthanized at day 15 and checked for absence of virus in their brains.

### Ethical Treatment of Animals

This study was performed in strict accordance with the French guidelines and recommendations on animal experimentation and welfare. The protocol was approved by the local Animal Ethics Committee (ANSES/ENVA/UPEC) (Permit Number: 15/02/11–13). Every effort was made to minimize suffering.

### Histology

Groups of 4 female outbred BALB/c mice, aged 6 weeks at the start of the experiment, were inoculated with 100 PFU of parental virus or infectious clones thereof or diluent alone i.p. Mice were euthanized at day 7 p.i and brains were recovered. Tissues were fixed in 10% neutral buffered formalin, dehydrated and embedded in paraffin. Tissue sections (4 µm) were prepared and stained with hematoxylin, eosin and saffron.

### Virulence in Embryonated Chicken Eggs

10-day-old pathogen-free chicken eggs (INRA, Jouy-en-Josas, France) were inoculated by the intravascular route. Eggs were inoculated with diluent alone (PBS, endotoxin free, pH 7.4 supplemented with 1 U/ml of penicillin and 1 µg/ml of streptomycin) or with either Kunjin, IS-98-ST1 parental or IC viruses. The eggs were incubated at 37°C and were observed regularly for up to 8 days. Viral RNA was analysed in the brain, heart, lungs, liver, intestines and kidneys after extraction using the RNeasy Kit (Qiagen), and amplified by quantitative RT-PCR as described above.

### Statistical Analysis

A Mann-Whitney test was applied to evaluate differences in survival curves. Paired T-test and One-way ANOVA were used to determine statistical significance for viral loads between groups infected with the parental or the IC derived viruses.

## Supporting Information

Figure S1 **Determination of the Lethal Dose 50 (LD50) of parental IS-98-ST1 virus in chicken embryo.** Groups of 6 ten-day-old pathogen-free chicken eggs were infected with 0.1, 1, 10 or 100 PFU of parental IS-98-ST1 virus. Eggs were monitored for 7 days post-infection.
(TIF)

## References

[1] Beasley DWC, Whiteman MC, Zhang S, Huang CYH, Schneider BS, et al (2005) Envelope protein glycosylation status influences mouse neuroinvasion phenotype of genetic lineage 1 west nile virus strains. J Virol 79 (13): 8339–8347. 10.1128/JVI.79.13.8339-8347.2005 PMC1143769 15956579

[2] Deas TS, Binduga-Gajewska I, Tilgner M, Ren P, Stein DA, et al (2005) Inhibition of flavivirus infections by antisense oligomers specifically suppressing viral translation and RNA replication. J Virol 79 (8): 4599–609. 10.1128/JVI.79.8.4599-4609.2005 PMC1069577 15795246

[3] Shi PY, Tilgner M, Lo MK, Kent KA, Bernard KA (2002) Infectious cDNA clone of the epidemic west nile virus from new york city. J virol 76 (12): 5847–5856. 10.1128/JVI.76.12.5847-5856.2002 PMC136194 12021317

[4] Samuel MA, Diamonds MS (2006) Pathogenesis of West Nile Virus infection: a balance between virulence, innate and adaptive immunity, and viral evasion. J Virol 80 (19): 9349–9360. 10.1128/JVI.01122-06 PMC1617273 16973541

[5] Puig-Basagoiti F, Tilgner M, Bennett CJ, Zhou Y, Mũnoz-Jordan JL, et al (2007) A mouse cell-adapted NS4B mutation attenuates West Nile virus RNA synthesis. Virology 361(1): 229–241. 17178141 10.1016/j.virol.2006.11.012 PMC1952232

[6] Melian EB, Hinzman E, Nagasaki T, Firth AE, Wills NM, et al (2010) NS1 of Flaviviruses in the Japanse Encephalitis Virus serogroup is a product of a ribosomal frameshifting and plays a role in viral neuroinvasiveness. J Virol 84 (3): 1641–1647. 10.1128/JVI.01979-09 PMC2812330 19906906

[7] Kummerer BM, Rice CM (2002) Mutations in the yellow fever virus nonstructural NS2A selectively block production of infectioux particles. J Virol 76 (10): 4773–4783. 10.1128/JVI.76.10.4773-4784.2002 PMC136122 11967294

[8] Liu WJ, Chen HB, Khromykh AA (2003) Molecular and functional analyses of Kunjin virus infectious cDNA clones demonstrate the essential roles for NS2A in virus assembly and for a nonconservative residue in NS3 in RNA replication. J Virol 77 (14): 7804–13. 10.1128/JVI.77.14.7804-7813.2003 PMC161959 12829820

[9] Liu W, Wang X, Mokhonov V, Shi P, Randall R, et al (2005) Inhibition of interferon signaling by the New York 99 strain and Kunjin subtype of West Nile Virus involves blockage of STAT1 and STAT2 activation by nonstructural proteins. J Virol 79(3): 1934–42. 15650219 10.1128/JVI.79.3.1934-1942.2005 PMC544092

[10] Mũnoz-Jordan JL, Sanchez-Burgos GG, Laurent-Rolle M, Garcia-Sastre A (2003) Inhibition of interferon signaling by dengue virus. Proc. Natl. Acad. Sci. USA 100 (24): 14333–14338. 10.1073/pnas.2335168100 PMC283592 14612562

[11] Pugachev K, Guirakhoo F, Ocran S, Mitchell F, Parsons M, et al (2004) High fidelity of yellow fever virus RNA polymerase. J Virol 78 (2): 1032–1038. 10.1128/JVI.78.2.1032-1038.2004 PMC368746 14694136

[12] May FJ, Davis CT, Tesh RB, Barrett ADT (2011) Phylogeography of West Nile Virus : from the Cradle of Evolution in Africa to Eurasia, Australia, and the Americas. J Virol. 85(6): 2964–2974. 10.1128/JVI.01963-10 PMC3067944 21159871

[13] Bondre VP, Jadi RS, Mishra AC, Yergolkar PN, Arankalle VA (2007) West Nile virus isolates from India : evidence for a distinct genetic lineage. J Gen Virol 88: 875–884. 17325360 10.1099/vir.0.82403-0

[14] Ma D, Jiang D, Qing M, Weidner JM, Qu X, et al (2009) Antiviral effect of interferon lambda against West Nile virus. Antiviral Res 83: 53–60. 19501257 10.1016/j.antiviral.2009.03.006 PMC2694136

[15] Calistri P, Giovannini A, Hubalek Z, Ionescu A, Monaco F, et al (2010) Epidemiology of West Nile in Europe and in the Mediterranean basin. The Open Virology J 4: 29–37. 20517490 10.2174/1874357901004010029 PMC2878979

[16] Papa A, Danis K, Baka A, Bakas A, Dougas G, et al (2010) Ongoing outbreak of West Nile virus infections in humans in Greece, July – August 2010. Eurosurveillance 15 (34): 1–5. 10.2807/ese.15.34.19644-en 20807489

[17] Beasley DWC, Li L, Suderman MT, Barrett ADT (2002) Mouse neuroinvasive phenotype of West Nile Virus strains varies depending upon virus genotype. Virology 296: 17–23. 12036314 10.1006/viro.2002.1372

[18] Botha EM, Markotter W, Wolfaardt M, Paweska JT, Swanepoel R, et al (2008) Genetic determinants of virulence in pathogenic lineage 2 West Nile Virus strains. Emerg Infect Dis 14(2): 222–230. 18258114 10.3201/eid1402.070457 PMC2600181

[19] Sotelo E, Gutierrez-Guzmán AV, del Amo J, Llorente F, El-Harrak M, et al (2011) Pathogenicity of two recent western Mediterranean West Nile virus isolates in a wild bird species indigenous to Southern Europe: the red-legged partridge. Vet Res 42: 11–19. 21314967 10.1186/1297-9716-42-11 PMC3037891

[20] Malkinson M, Banet C, Weisman Y, Pokamunski S, King R, et al (2002) Introduction of West Nile virus in the Middle East by migrating white storks. Emerg Infect Dis 8 392–397. 11971773 10.3201/eid0804.010217 PMC2730252

[21] Mashimo T, Lucas M, Simon-Chazottes D, Frenkiel MP, Montagutelli X, et al (2002) A nonsense mutation in the gene encoding 2′-5′-oligoadenylate synthetase/L1 isoform is associated with West Nile virus susceptibility in laboratory mice. Proc. Natl. Acad. Sci. USA 99 (17): 11311–11316. 10.1073/pnas.172195399 PMC123253 12186974

[22] Lucas M, Frenkiel M-P, Mashimo T, Guénet J-L, Deubel V, et al (2004) The israeli strain IS-98-ST1 of West Nile virus as viral model for West Nile encephalitis in the Old world. Virol J 18: 1–9. 10.1186/1743-422X-1-9 PMC535539 15550172

[23] Mertens E, Kajaste-Rudnitski A, Torres S, Funk A, Frenkiel M-P, et al (2010) Viral determinants in the NS3 helicase and 2K peptide that promote West Nile virus resistance to antiviral action of 2′, 5′ oligoadenylate synthase 1 b. Virology 399(1): 176–185. 20100623 10.1016/j.virol.2009.12.036

[24] Kajaste-Rudnitski A, Mashimo T, Frenkiel MP, Guénet JL, Lucas M, et al (2006) The 2′, 5′-Oligoandénylate Synthetase 1 b Is a Potent Inhibitor of West Nile Virus Replication Inside Infected Cells. J Biol Chem 281(6): 4624–4637. 16371364 10.1074/jbc.M508649200

[25] Simon-Chazotte D, Frenkiel M-P, Montagutelli X, Guénet J-L, Desprès P, et al (2011) Transgenic expression of full length 2′,5′-oligoadenylate synthase 1 b confers to BALB/c mice resistance against West Nile virus-induced encephalitis. Virology 417: 147–153. 21683973 10.1016/j.virol.2011.05.018

[26] Yamschchikov VF, Wengler G, Peregylin AA, Brinton MA, Compans R (2001) An infectious clone of the west nile flavivirus. Virology 281, 294–304. 10.1006/viro.2000.0795 11277701

[27] Evans JD, Seeger C (2007) Differential effects of mutations in NS4B on West Nile Virus replication and inhibition of interferon signaling. J Virol 81(21): 11809–11816. 17715229 10.1128/JVI.00791-07 PMC2168815

[28] Khromykh AA, Westaway EG (1997) Subgenomic replicons of the flavivirus Kunjin: construction and applications. J Virol 71(2): 1497–505. 8995675 10.1128/jvi.71.2.1497-1505.1997 PMC191206

[29] Murray KO, Mertens E, Desprès P (2010) West Nile virus and its emergence in the United States of America. Vet Res 41 (6): 67–81. 10.1051/vetres/2010039 PMC2913730 21188801

[30] Kinney RM, Huang CYH, Whiteman MC, Bowen RA, Langevin SA, et al (2006) Avian virulence and thermostable replication of the North American strain of West Nile virus. J Gen Virol 87: 3611–3622. 17098976 10.1099/vir.0.82299-0

[31] Crespo R, Shivaprasad HL, França M, Woolcock PR (2009) Isolation and Distribution of West Nile Virus in Embryonated Chicken Eggs. Avian Diseases 53: 608–612. 20095164 10.1637/8829-040209-ResNote.1

[32] Senne DA, Pedersen JC, Hutto DL, Taylor WD, Schmitt BJ, et al (2000) Pathogenicity of West Nile virus in chickens. Avian Dis 44 (3): 642–649. 11007013

[33] Steel KE, Linn MJ, Schoepp RJ, Komar N, Geisbert TW, et al (2000) Pathology of Fatal West Nile Virus Infections in Native and Exotic Birds during the 1999 Outbreak in New York City, New York. Vet Pathol 37: 208–224. 10810985 10.1354/vp.37-3-208

[34] Hall RA, Broom AK, Smith DW, Mackenzie JS (2002) The ecology and epidemiology of Kunjin virus. Curr.Top.Microbiol. Immunol 267: 253–269. 10.1007/978-3-642-59403-8_13 12082993

[35] Seregin A, Nistler R, Borisevich V, Yamshchikov G, Chaporgina E, et al (2006) Immunogenicity of west nile virus infectious DNA and its noninfectious derivatives. Virology 356: 115–125. 16935318 10.1016/j.virol.2006.07.038

[36] Borisevich V, Seregin A, Nistler R, Mutabazi D, Yamshchikov V (2006) Biological properties of chimeric West Nile viruses. Virology 349: 371–381. 16545851 10.1016/j.virol.2006.02.013

[37] Ruggli N, Rice CM (1999) Functional cDNA clones of the Flaviviridae: strategies and applications. Adv Virus Res 53: 183–207. 10582099 10.1016/s0065-3527(08)60348-6

[38] Yamshchikov V, Mishin V, Cominelli F (2001) A new strategy in design of +RNA virus infectious clones enabling their stable propagation in E. coli. Virology 281(2): 272–80. 11277699 10.1006/viro.2000.0793

[39] Elghonemy S, Davis WG, Brinton MA (2005) The majority of the nucleotides in the top loop of the genomic 3′terminal stem loop structure are cis-acting in a West Nile virus infectious clone. Virology 331: 238–246. 15629768 10.1016/j.virol.2004.11.008

[40] Khromykh AA, Westaway EG (1994) Completion of Kunjin virus RNA sequence and recovery of an infectious RNA transcribed from stably cloned full-length cDNA. J Virol 68: 4580–4588. 8207832 10.1128/jvi.68.7.4580-4588.1994 PMC236385

[41] Fodor E, Devenish L, Engelhardt OG, Palese P, Brownlee GG, et al (1999) Rescue of influenza A virus from recombinant DNA. J Virol 73: 9679–9682. 10516084 10.1128/jvi.73.11.9679-9682.1999 PMC113010

[42] Yamshchikov G, Borisevich V, Seregin A, Chaporgina E, Mishina M, et al (2004) An attenuated West Nile prototype virus is highly immunogenic and protects against the deadly NY99 strain: a candidate for live WN vaccine development. Virology 330(1): 304–12. 15527855 10.1016/j.virol.2004.09.014

[43] Audsley M, Edmonds J, Liu W, Mokhonov V, Mokhonova E, et al (2011) Virulence determinants between New York 99 and Kunjin strains of West Nile Virus. Virology 414 (1): 63–73. 10.1016/j.virol.2011.03.008 PMC3089702 21477835

[44] Firth AE, Wills NM, Gesteland RF, Atkins JF (2011) Stimulation of stop codon readthrough: frequent presence of an extended 3′RNA structural element. Nucleic Acids Res 39(15): 6679–6691. 21525127 10.1093/nar/gkr224 PMC3159437

[45] Firth AE, Brierley I (2012) Non-canonical translation in RNA viruses. J Gen Virol 93(Pt 7): 1385–1409. 10.1099/vir.0.042499-0 PMC3542737 22535777

[46] Jungreis I, Lin MF, Spokony R, Chan CS, Negre N, et al (2011) Evidence of abundant stop codon readthrough in Drosophila and other metazoan. Genome Res 21: 2096–2113. 21994247 10.1101/gr.119974.110 PMC3227100

[47] Abdul-Careem MF, Hunter DB, Lambourne MD, Barta J, Sharif S (2007) Ontogeny of cytokine gene expression in the chicken spleen. Poult Sci. 86(7): 1351–1355. 10.1093/ps/86.7.1351 17575181

[48] Jacobsen ID, Grosse K, Slesiona S, Hube B, Berndt A, et al (2010) Embryonated eggs as an alternative infection model to investigate Aspergillus fumigates virulence. Infect. Immun. 78(7): 2995–3006. 10.1128/IAI.00268-10 PMC2897382 20421382

[49] Liang QL, Luo J, Zhou K, Dong JX, He HX (2011) Immune-related gene expression in response to H5N1 avian influenza virus infection in chicken and duck embryonic fibroblasts. Mol Immunol 48(6–7): 924–30. 21256597 10.1016/j.molimm.2010.12.011

[50] Schlick P, Taucher C, Schittl B, Tran JL, Kofler RM, et al (2009) Helices α2 and α3 of West Nile Virus capsid protein are dispensable for assembly of infectious virions. J Virol 83 (11): 5581–5591. 10.1128/JVI.02653-08 PMC2681950 19297470

[51] Yu L, Putnak JR, Pletnev AG, Markoff L (2008) Attenuated West Nile viruses bearing 3′SL and envelope gene substitution mutations. Vaccine 26 (47): 5981–5988. 10.1016/j.vaccine.2008.08.064 18805457

[52] Liu WJ, Wang XJ, Clark DC, Lobigs M, Hall RA, et al (2006) A single amino acid substitution in the West Nile nonstructural protein NS2A disables its ability to inhibit alpha/beta interferon induction and attenuates virus virulence in mice. J Virol 80 (5): 2396–2404. 10.1128/JVI.80.5.2396-2404.2006 PMC1395377 16474146

[53] Wicker JA, Whiteman MC, Beasley DWC, Todd Davis C, Zhang S, et al (2006) A single amino acid substitution in the central portion of the West Nile NS4B protein confers a highly attenuated phenotype in mice. Virology 349(2): 245–253. 16624366 10.1016/j.virol.2006.03.007

[54] Brault AC, Huang CY-H, Langevin SA, Kinney RM, Bowen RA, et al (2007) A single positively selected West Nile viral mutation confers increased virogenesis in American crows. Nat Genet 39 (9): 1162–1166. 10.1038/ng2097 PMC2291521 17694056

[55] Sotelo E, Fernandez-Pinero J, Llorente F, Agüero M, Hoefle U, et al (2009) Characterization of West Nile Virus isolates from Spain : New insights into the distinct West Nile Virus eco-epidemiology in the Western Mediterranean. Virology 395: 289–297. 19833373 10.1016/j.virol.2009.09.013

[56] Sambrook J, Fritsch EF, Maniatis T (1989) Molecular cloning : a laboratory manual, 2nd ed. Cold Spring Harbor, NY: Cold Spring Harbor Laboratory Press.

[57] Sambrook J, Russel DW (2001) Molecular cloning, 3rd ed. Cold Spring Harbor, NY: Cold Spring Harbor Laboratory Press.

[58] Brnic D, Stevanovic V, Cochet M, Agier C, Richardson J, et al (2012) Borna Disease Virus Infects Human Neural Progenitor Cells and Impairs Neurogenesis. J Virol 86(5): 2512–2522. 22190725 10.1128/JVI.05663-11 PMC3302287

[59] Linke S, Ellerbrok H, Niedrig M, Nitsche A, Pauli G (2007) Detection of west nile virus lineage 1 and 2 by real-time PCR. J Virol Meth 146: 355–358. 10.1016/j.jviromet.2007.05.021 17604132

