# Supplementary figures and images for: IS-98-ST1 West Nile Virus Derived from an Infectious cDNA Clone Retains Neuroinvasiveness and Neurovirulence Properties of the Original Virus
Source: PLoS One. 2012 Oct 23;7(10):e47666. doi: 10.1371/journal.pone.0047666 (PMC3479121; doi:10.1371/journal.pone.0047666)

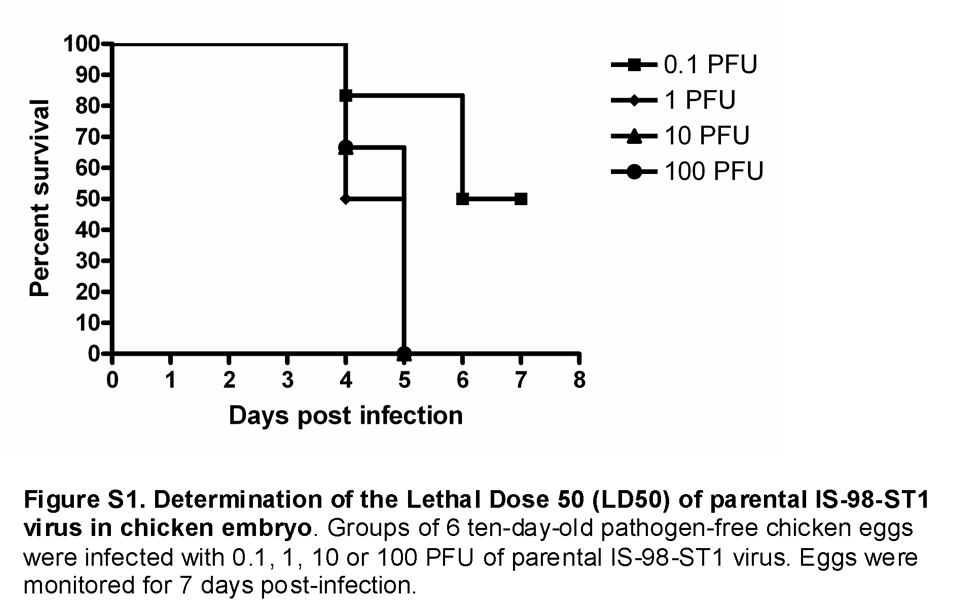

Supplement: Figure S1 — Determination of the Lethal Dose 50 (LD50) of parental IS-98-ST1 virus in chicken embryo. Groups of 6 ten-day-old pathogen-free chicken eggs were infected with 0.1, 1, 10 or 100 PFU of parental IS-98-ST1 virus. Eggs were monitored for 7 days post-infection. (TIF) [file pone.0047666.s001.tif]
